# Prognostic signatures of sphingolipids: Understanding the immune landscape and predictive role in immunotherapy response and outcomes of hepatocellular carcinoma

**DOI:** 10.3389/fimmu.2023.1153423

**Published:** 2023-03-17

**Authors:** Xin Zhang, Jinke Zhuge, Jinhui Liu, Zhijia Xia, Huixiong Wang, Qiang Gao, Hao Jiang, Yanyu Qu, Linlin Fan, Jiali Ma, Chunhua Tan, Wei Luo, Yong Luo

**Affiliations:** ^1^ Department of Pathology, the Second People’s Hospital of Foshan, Affiliated Foshan Hospital of Southern Medical University, Foshan, China; ^2^ Department of Respiratory Medicine, Hainan Cancer Hospital, Hainan, China; ^3^ Department of Gynecology, The First Affiliated Hospital of Nanjing Medical University, Nanjing, China; ^4^ Department of General, Visceral, and Transplant Surgery, Ludwig-Maximilians-University Munich, Munich, Germany; ^5^ Department of Hepatobiliary Surgery, Hospital of Inner Mongolia Baotou Steel, Baotou, Inner Mongolia, China; ^6^ Department of Hepatobiliary Surgery, The First Affiliated Hospital of Guangxi Medical University, Nanning, China; ^7^ Department of General Surgery, The Affiliated Traditional Chinese Medicine Hospital of Southwest Medical University, Luzhou, China; ^8^ Department of Urology, The Second People’s Hospital of Foshan, Affiliated Foshan Hospital of Southern Medical University, Foshan, China

**Keywords:** HCC, immune, sphingolipid, immunotherapy response, prediction

## Abstract

**Background:**

Hepatocellular carcinoma (HCC) is a complex disease with a poor outlook for patients in advanced stages. Immune cells play an important role in the progression of HCC. The metabolism of sphingolipids functions in both tumor growth and immune infiltration. However, little research has focused on using sphingolipid factors to predict HCC prognosis. This study aimed to identify the key sphingolipids genes (SPGs) in HCC and develop a reliable prognostic model based on these genes.

**Methods:**

The TCGA, GEO, and ICGC datasets were grouped using SPGs obtained from the InnateDB portal. A prognostic gene signature was created by applying LASSO-Cox analysis and evaluating it with Cox regression. The validity of the signature was verified using ICGC and GEO datasets. The tumor microenvironment (TME) was examined using ESTIMATE and CIBERSORT, and potential therapeutic targets were identified through machine learning. Single-cell sequencing was used to examine the distribution of signature genes in cells within the TME. Cell viability and migration were tested to confirm the role of the key SPGs.

**Results:**

We identified 28 SPGs that have an impact on survival. Using clinicopathological features and 6 genes, we developed a nomogram for HCC. The high- and low-risk groups were found to have distinct immune characteristics and response to drugs. Unlike CD8 T cells, M0 and M2 macrophages were found to be highly infiltrated in the TME of the high-risk subgroup. High levels of SPGs were found to be a good indicator of response to immunotherapy. In cell function experiments, SMPD2 and CSTA were found to enhance survival and migration of Huh7 cells, while silencing these genes increased the sensitivity of Huh7 cells to lapatinib.

**Conclusion:**

The study presents a six-gene signature and a nomogram that can aid clinicians in choosing personalized treatments for HCC patients. Furthermore, it uncovers the connection between sphingolipid-related genes and the immune microenvironment, offering a novel approach for immunotherapy. By focusing on crucial sphingolipid genes like SMPD2 and CSTA, the efficacy of anti-tumor therapy can be increased in HCC cells.

## 1 Introduction

Hepatocellular carcinoma (HCC) is the most prevalent type of primary liver cancer worldwide, accounting for 90% of cases (1). This disease is often found in individuals with cirrhosis and can be caused by various environmental factors, such as tobacco and aflatoxin, as well as etiologies such as HBV infection, alcohol consumption, and non-alcoholic steatohepatitis (2–4). Like pancreatic cancer, inflammation is also a significant contributor to the development of HCC. HCC develops from dysplastic nodules and progresses through a series of histopathological stages. Despite surgical resection, patients with HCC often face poor prognoses due to the high degree of heterogeneity within patients and even within individual tumors, which leads to drug resistance and recurrence (5–7). Checkpoint inhibitor immunotherapy has demonstrated potent anti-tumor effects in a subset of cancer patients (8–10). Hepatocellular carcinoma (HCC) is also known to be regulated by the immune system. The combination of the anti-PDL1 antibody atezolizumab and the VEGF-neutralizing antibody bevacizumab has been proposed as a first-line therapy for HCC, and is currently undergoing clinical trials (11). However, a major challenge for HCC checkpoint immunotherapy is the identification and validation of reliable predictive biomarkers. To improve treatment outcomes, new biomarkers that can predict patient outcomes are needed.

Sphingolipids, structural molecules found in cell membranes, play a crucial role in regulating various biological processes, including growth, proliferation, migration, invasion, and metastasis in cancer (12). As second signaling molecules, they also control programmed cell death, cell differentiation, aging, and growth. The key components of sphingolipids are sphingomyelin, ceramide, sphingosine-1-phosphate, sphingomyelin, and glycosphingolipids (13). Alterations in sphingolipid synthesis can affect various signaling pathways, promoting or inhibiting tumor progression (14–17). Recent studies have shown that certain members of the sphingolipid class are linked to the development of HCC and have a prognostic value (18, 19). Despite the established importance of sphingolipids in HCC, few studies have systematically evaluated the potential of sphingolipid-associated genes (SPGs) in predicting prognosis. A deeper understanding of these genes can lead to improved survival rates and treatment responses.

Our study aimed to create a prognostic model utilizing sphingolipid-associated genes (SPGs) from the TCGA-LIHC cohort, which we then combined with clinicopathological characteristics to build a nomogram for predicting prognosis and providing clinical treatment guidance. The nomogram’s clinical prognostic value was verified through time-dependent ROC and DCA curve analysis. Our findings suggest that sphingolipid-associated genes have the potential to predict the prognosis of HCC patients and provide new, experimentally validated biomarkers for precision targeted therapy.

## 2 Materials and methods

### 2.1 Data acquisition

In our study, we obtained gene expression profiles and clinical data, such as TNM classification, age, gender, and overall survival, from the TCGA-LIHC cohort (including 374 LIHC and 50 normal tissue samples) on the TCGA data portal (https://portal.gdc.cancer.gov/). We also downloaded the GSE14520 dataset, which contained 221 HCC samples, from the GEO database (https://www.ncbi.nlm.nih.gov/geo/) and the ICGC dataset, which contained 240 HCC samples, from https://icgc.org/. Only data that had complete clinical information was used for analysis. We normalized the transcripts per million (TPM) data and then applied a log2 transformation (20).

### 2.2 Access to sphingolipid-associated genes

We utilized the InnateDB portal, a publicly available database of genes, proteins, and experimentally validated interactions, to gather a set of 97 sphingolipid-associated genes (SPGs). This database currently contains 18,780 curated interactions, allowing for easy querying of various gene sets. The SPGs were downloaded directly from the InnateDB portal (http://www.innatedb.com) (21).

### 2.3 Consensus clustering

To investigate the involvement of sphingolipid-associated genes in HCC, a set of 97 SPGs with a statistical significance of *P* < 0.01 were subjected to Consensus Clustering using the ‘ConsensusClusterPlus’ R package (22). The HCC cohort was classified into two distinct groups (k = 2) based on this analysis. To validate the reliability of the clustering, we then applied the UMAP method using the package “ggplot2” in R.

### 2.4 GSVA analysis

To analyze the functional significance of the identified SPGs, we used the file “c2.cp.kegg.v7.4.symbols.gmt” from the MSigDB database and performed GSVA enrichment analysis using the “GSVA” R package (23–25)

. We applied the “limma” R package to adjust for P values (*P* < 0.05) and determine statistical significance of subgroup differences (26, 27). Additionally, we used functional enrichment analysis to investigate the pathways and functional annotations associated with DEGs related to SPGs in HCC. We also used the “heatmap” R package to create a visual representation of the data.

### 2.5 LASSO regression analysis

In this study, we used univariate Cox regression analysis to identify a total of 28 sphingolipid-associated genes (SPGs) that were associated with the survival of HCC patients. Next, we employed LASSO regression analysis using the “glmnet” R package and determined the parameter λ through tenfold cross-validation. Finally, we selected 6 core genes through a multivariate Cox regression model. We used the best lambda scores and coefficients to construct a 6-SPG risk signature. The risk score for each patient was calculated as follows: risk score = e^(… corresponding coefficient +… + SELL expression). Overall, this study identified and validated 6-SPGs associated with prognosis in the TCGA-LIHC, GSE14520, and ICGC cohort using univariate Cox regression analysis.

### 2.6 Immune cell Infiltration

To evaluate the infiltration of immune cells in our samples, we employed both CIBERSORT and ssGSEA R scripts (28, 29). By utilizing CIBERSORT, we calculated a score for each sample reflecting the estimated proportion of immune cell types present. These scores were then used to compare the distribution of low-risk and high-risk immune cell types. We also performed a spearman rank correlation analysis to investigate the relationship between our calculated risk scores and the presence of different immune cells.

### 2.7 Nomogram

We used clinicopathological characteristics and risk scores to create nomograms, which are diagrams that predict the likelihood of a certain outcome. To ensure accuracy, we conducted a calibration plot. Additionally, we used decision curve analysis (DCA) to evaluate the clinical utility of these nomograms (30, 31).

### 2.8 Predicting chemotherapy response

QuartataWeb (http://quartata.csb.pitt.edu) is a user-friendly server that allows users to analyze drugs and genomics. The platform allows easy access to the DrugBank and STITCH databases, which facilitate the exploration of protein-drug and protein-chemical interactions (32). Additionally, the “pRRophetic” R package was utilized to calculate the median inhibitory concentration (IC50) of small molecule drugs.

### 2.9 Cancer cell line encyclopedia (CCLE)

We obtained the mRNA expression matrix for tumor cell lines from the CCLE dataset (33). The data was visualized using the “ggplot2” package in R v4.1.3.

### 2.10 Human protein atlas (HPA)

The HPA portal (Human Protein Atlas proteinatlas.org) is a valuable resource for researchers, providing immunohistochemical (IHC) data for proteins found in all major human tissues. The portal also allows users to view the subcellular localization of proteins in individual cells (34).

### 2.11 Cell culture

The Huh7 HCC cell line was obtained from the ATCC company and cultured at 37°C in a 5% CO2 atmosphere in DMEM (Thermo Scientific HyClone) supplemented with 10% fetal bovine serum (Gibco FBS). Lipofectamine™ 3000, the CCK-8 assay kit, and Lapatinib were purchased from Invitrogen, Dojindo, and MedChemExpress, respectively.

### 2.12 RNA transfection

Huh7 cells were transfected with siRNA specific for CSTA (sc-44430, Santa Cruz), SMPD2 (sc-106277, Santa Cruz), and a control siRNA (sc-37007, Santa Cruz) using Lipofectamine 3000 (Thermo Fisher Scientific) for a duration of 48 hours

### 2.13 RNA Extraction and real-time PCR

Before beginning the real-time PCR process, we briefly centrifuged the samples and used 1 microgram of total RNA as the template. We followed the instructions provided by the FastKing One Step RT-qPCR kit (SYBR Green) (TIANGEN, Beijing, China) and ran the standard PCR reaction program as follows: the reverse transcription step was performed at 50 Celsius for 30 minutes, followed by an initial denaturation step at 95 Celsius for 3 minutes and 40 consecutive cycles of 15 seconds at 95 Celsius and 30 seconds at 60 Celsius. The sequences for the CSTA, SMPD2, and GAPDH primers can be found in the **Supplementary Figure S1**.

### 2.14 Cell viability and wound-healing migration assay

The cell viability of Huh7 cells was determined using the Cell Count Kit-8 (Dojindo, Japan). The CCK8 reagent (10 μl) was added to each well and the cells were incubated at 37°C in 5% CO2 for 1.5 hours. The optical density (OD) value at 450 nm was then measured. The migration ability of the Huh7 cells was also assessed after being treated with siRNA for 48 hours. The photographs were taken at the same location at both 48 hours and 0 hours after scratching.

### 2.15 TISCH database and best database

The Tumor Immune Single-Cell Hub (35) (TISCH) acts as a web-based resource for single-cell RNA-Seq data pertaining to the tumor microenvironment (TME). By utilizing this tool, we were able to investigate the distribution of 6-SPGs among various cell types within the microenvironment of hepatocellular carcinoma (HCC). Additionally, we utilized the “BEST” analytical pipeline available on the website “rookieutopia.com” to analyze cancer biomarkers and predict immunotherapy outcomes by examining different subgroups of risk.

### 2.16 Statistical analysis

We utilized R version 4.1.3 for statistical analysis. The survival rates of the two groups were compared using Kaplan-Meier curves and a log-rank test, with the aid of the R package “survminer” to generate the survival curves. Additionally, LASSO-Cox analysis was applied to construct prognostic gene signatures following Cox regression evaluation. The groups were divided into high- and low-risk based on the Wilcox test for immune function and tumor-infiltrating immune cells. Results were considered statistically significant if the p-value was less than 0.05 and the false discovery rate (FDR) q was less than 0.05. All experimental data was presented as the means ± SEM. Statistical analysis was performed using Student’s t-test for comparisons between two groups or one-way ANOVA with Tukey *post hoc* test for multiple comparisons. Statistical analysis was conducted using GraphPad Prism 8 (GraphPad Software, USA). Significance was determined as *P* < 0.05. * denotes *P* < 0.05, ** denotes *P* < 0.01, *** denotes *P* < 0.001.

## 3 Results

### 3.1 Identification of prognostically associated SPGs

**Figure 1:**
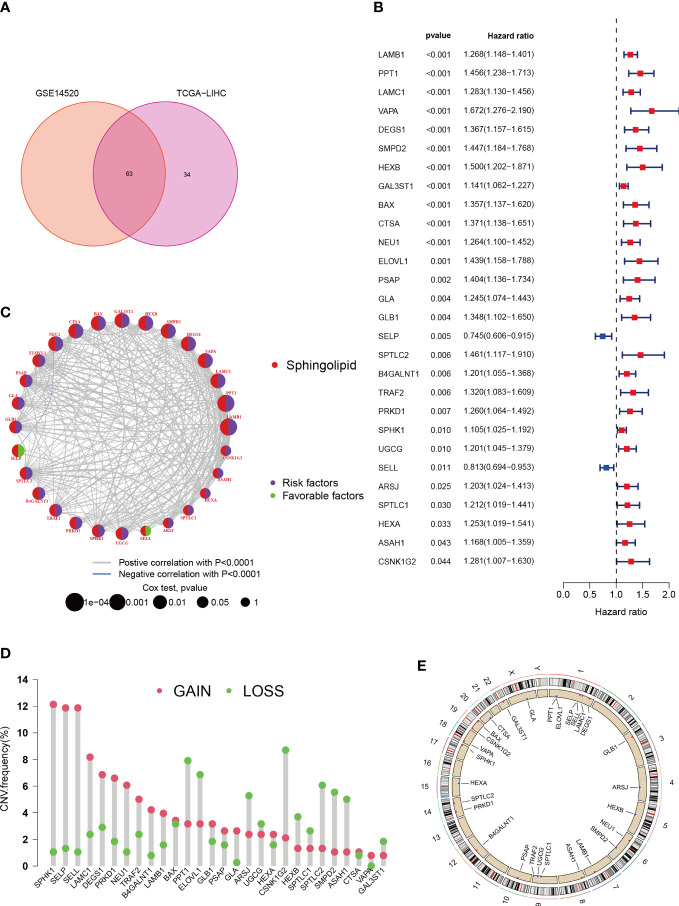
Characteristics and differences of sphingolipid-associated genes in HCC. **(A)** 63 sphingolipid-associated genes identified from TCGA-LIHC and GSE14520 cohort. **(B)** 28 SPGs (*P* < 0.05) *via* the univariate Cox regression analysis. **(C)** Linkage between the 28 SPGs. **(D)** CNVs of 28 SPGs in TCGA-LIHC. **(E)** Chromosome site of SPGs.

By merging data from TCGA-HCC and GSE14520, we were able to eliminate batch effects and create the “LIHC-GSE14520” cohort, which contained 14,490 genes. We then sourced 97 SPGs from the InnateDB portal and used a Venn plot to identify 63 SPGs present in both the TCGA-LIHC and GSE14520 cohorts (**Figure 1A**). We then compared these SPGs to normal adjacent tissue samples and identified 49 DEGs in HCC samples. We conducted a univariate Cox regression analysis on these 49 SPGs and found that 28 were significantly associated with survival (*P* < 0.05, km < 0.05), and 26 of these, excluding SELP and SELL, were associated with poor prognosis (**Figure 1B**). Additionally, a network plot was created to better understand the relationships between these 28 SPGs (**Figure 1C**). We also analyzed CNV data from the TCGA portal, as chromosomal alterations are a common feature of tumors (36). We examined the location of each SPG on the chromosome and the extent to which they were altered (**Figures 1D, E**). **Figures 1D, E** shows that the most notable “gains” and “losses” occurred on chromosome 17 with SPHK1 and on chromosome 19 with CSNK1G2.

### 3.2 Consistent clustering

**Figure 2:**
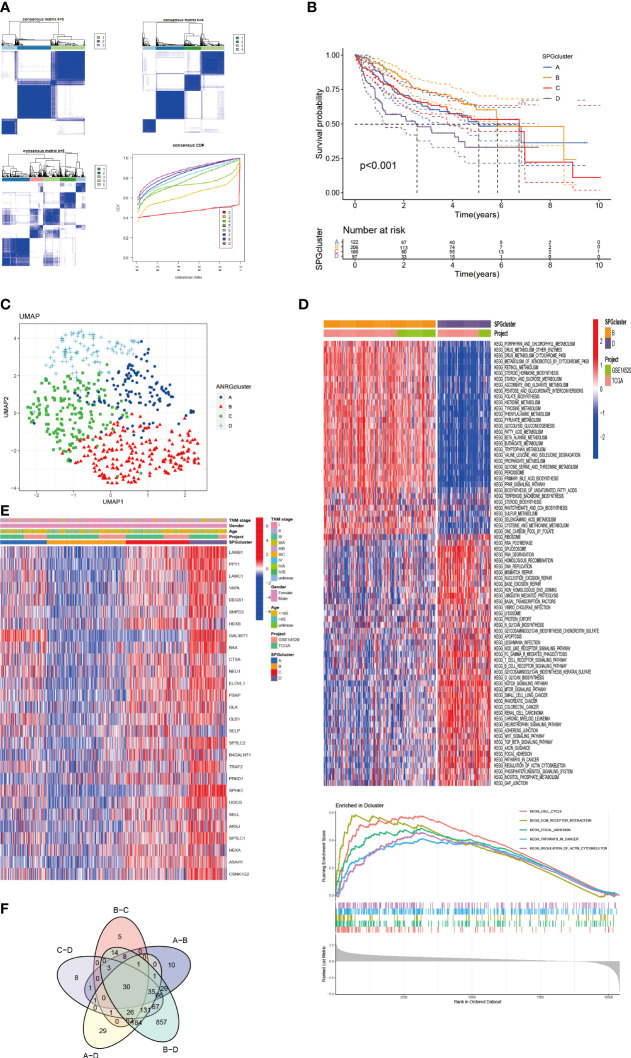
HCC Subgroups classified by SPGs. **(A)** Consensus matrix for k = 4 was accepted using consensus clustering. **(B)** Survival probability of 4 subtypes (*P* < 0.01). **(C)** UMAP distinguished 4 subtypes based on SPGs expression level. **(D)** Clusters D and B were analyzed differently using GSVA in terms of KEGG pathway enrichment. **(E)** Clinical and pathological features of four subtypes of SPGs expression. **(F)** Differential SPGs-based clusters intersected on Venn plots.

To gain a deeper understanding of the role of 28 specific proteins in HCC, we utilized Consensus Clustering with the “ConsensusClusterPlus” R package. Our results, shown in **Figure 2A**, revealed a well-defined grouping of the cohort when k = 4. Furthermore, Kaplan-Meier survival curves indicated that these four clusters had significant differences in overall survival (*P* < 0.001) as shown in **Figure 2B**. The UMAP analysis also confirmed the correct assignment of the four clustering subtypes at k=4, as shown in **Figure 2C**. Additionally, we examined the correlation between clinicopathological features and the expression of the 28 specific proteins in the four clusters. The heat map in **Figure 2E** revealed that LAMB1 and SPHK1 were notably present in cluster D, suggesting their potential involvement in tumor progression. To further investigate the differential expression of these 28 specific proteins in the subtypes, we applied the GSVA package for KEGG pathway enrichment analysis between cluster D and B, which showed a significant difference in survival between them as shown in **Figure 2D**. Cluster D, associated with the worst prognosis, was found to mainly involve the Cell Cycle, Focal Adhesion, ECM Receptor Interaction, and other pathways in cancer, which may partially explain the poor survival. Finally, the Venn diagrams in **Figure 2F** illustrated the distinct distribution of differentially expressed genes within the four subtypes.

### 3.3 Immune infiltration in the subtypes

**Figure 3:**
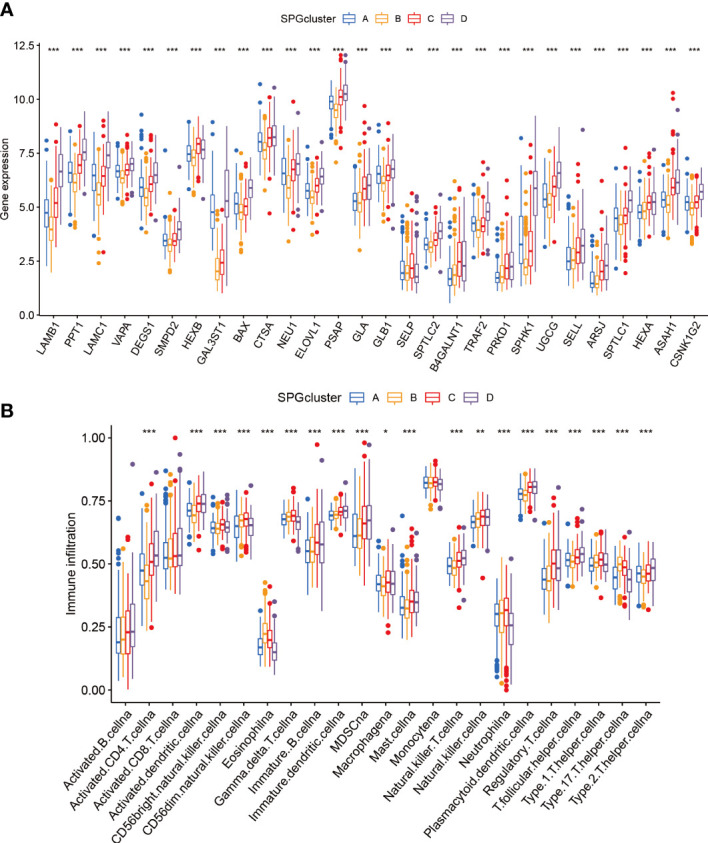
Patterns of immune infiltration and gene expression among four SPGs-based clusters. **(A)** SPGs expression of four subtypes. **(B)** Immune infiltration patterns of four subtypes. (Wilcox test, *P < 0.05, **P < 0.01, ***P < 0.001).

We used boxplots to illustrate the expression patterns of the 28 SPGs across the four subgroups. As shown in **Figure 3A**, most SPGs had high expression levels in subgroup D, with the exception of SELP. Notably, a previous study found that high expression of the SELL gene was linked to a positive prognosis. We therefore suggest that SELL may be a crucial sphingolipid gene involved in the body’s natural defense against tumor growth. Given that subgroup D had a poor prognosis, these SPGs may play a role in HCC progression and may be potential targets for HCC-specific treatments. Additionally, we observed notable variations in immune cell infiltration among the subtypes (**Figure 3B**; **Supplementary Figure S2**). Subgroup D had higher levels of immune infiltration, including MDSC cells with immunosuppressive properties, indicating that SPGs may contribute to an immunosuppressive microenvironment.

### 3.4 SPGs signature construction

**Figure 4:**
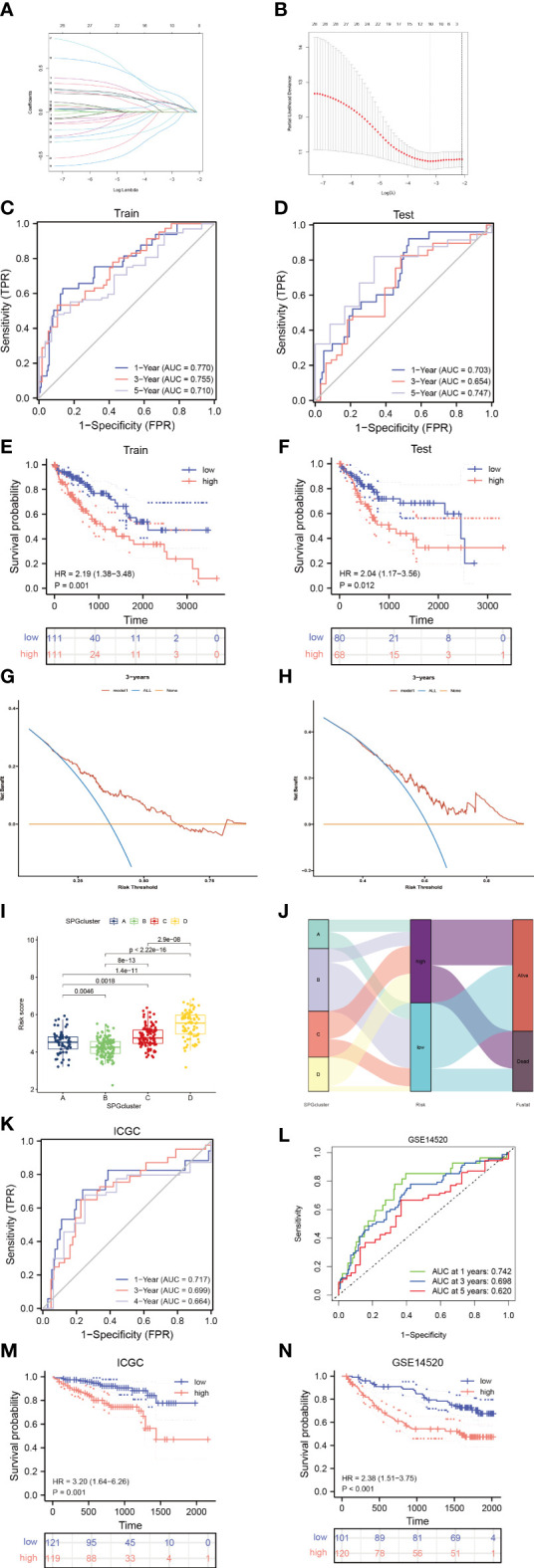
Identify sphingolipid-associated predictive signature. **(A, B)** LASSO analysis identified 6 prognostic SPGs. **(C, D)** The time-dependent ROC exhibited the sensitivity and specificity of constructed riskscore model. **(E, F)** Survival probability of differential risk subgroups. **(G, H)** Decision curve analysis of 6-SPGs riskscore model for predicting survival status, including OS and PFS. **(I)** Risk score in 4 SPGclusters. **(J)** Alluvial diagram of SPGcluster and associated living status. **(K, L)** Validity of the model in the external validation sets. **(M, N)** Survival probability of differential risk subgroups in external validation cohorts.

To create a model that accurately predicts the risk for each patient, we divided the samples in the “TCGA-LIHC” cohort into two groups: a training group and a test group. We applied LASSO-Cox regression analysis to the differentially expressed genes in the training group and identified 6 signature genes associated with risk (**Figures 4A, B**). We named this risk score “riskscore”, and the coefficients for each signature gene are listed in **Supplementary Table 1**. The time-dependent ROC curves for overall survival at 1 and 3 years in both the train/test group and validation groups (GSE14520, ICGC cohort) showed strong performance in predicting overall survival (**Figures 4C, D, K, L**). Furthermore, a significant survival advantage was observed in the low-risk group compared to the high-risk group (*P* < 0.05) (**Figures 4E, F, M, N**). The DCA curve indicated that this risk model is useful for clinical application and could improve overall survival and progression-free survival for patients with HCC (**Figures 4G, H**). As shown in **Figures 4I, J**, the risk scores varied significantly across four subtypes related to the signature genes. We also described the link between the signature gene clusters, riskscore, and living status in an alluvial diagram.

### 3.5 Immune infiltration

**Figure 5:**
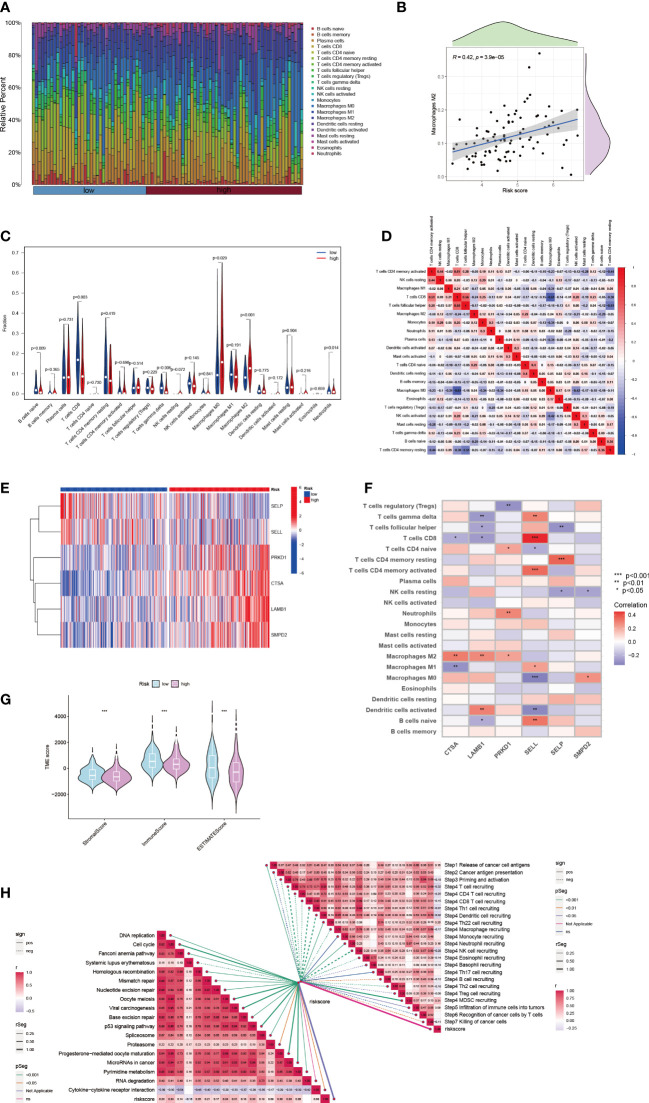
HCC immune microenvironment at differential risk score. **(A)** Risk score associated with different proportions of infiltrating immune cells. **(B)** Correlation between risk score and Macrophage M0 cells in HCC tissues. **(C)** A comparison of immune cell components between high-risk and low-risk groups. **(D)** Correlation between immune cells. **(E)** Expression patterns of 6-SPGs. **(F)** Correlation between immune cells and 6-SPGs. **(G)** Estimate score of the risk subgroups. **(H)** Correlation between riskscore and cancer-immunity cycle as well as functional pathways. (Wilcox test, *P < 0.05, **P < 0.01, ***P < 0.001).

The microenvironment of a tumor, specifically the immune system, plays a critical role in the development of tumors. When the immune system is not functioning properly, it allows for tumor cells to evade immune surveillance (37, 38). To quantify the landscape of the microenvironment in patients with high and low risk of HCC, we used the CIBERSORT R script. We first ranked patients according to their risk score and calculated the proportion of different immune cells present in each patient (**Figure 5A**). We found a significant correlation between the proportion of Macrophage M2 cells and the risk score (R = 0.42) (**Figure 5B**). Additionally, in HCC tissue, the majority of immune cells present were macrophages M0 and M2 (**Figure 5C**), indicating that these macrophages likely play a significant role in the development of HCC (39). We also observed a negative correlation between macrophages M0 and CD8 T cells in the microenvironment of HCC tissue (R = -0.61) (**Figure 5D**). The 6-SPGs signature that was used to construct the risk score model had different expression patterns and was strongly correlated with multiple immune cell infiltrations. CD8 T cells were positively correlated with SELL, while the opposite relationship was observed with CTSA and LAMB1 (**Figures 5E, F**). Furthermore, we calculated stromal, immune, and tumor scores in patients with different risk levels using the “estimate” R package for expression profiles (**Figure 5G**). Overall, the high-risk group had a weaker immune response, in addition to increased tumor proliferation and DNA replication (**Figure 5H**).

### 3.6 Nomogram predicts HCC patients survival

**Figure 6:**
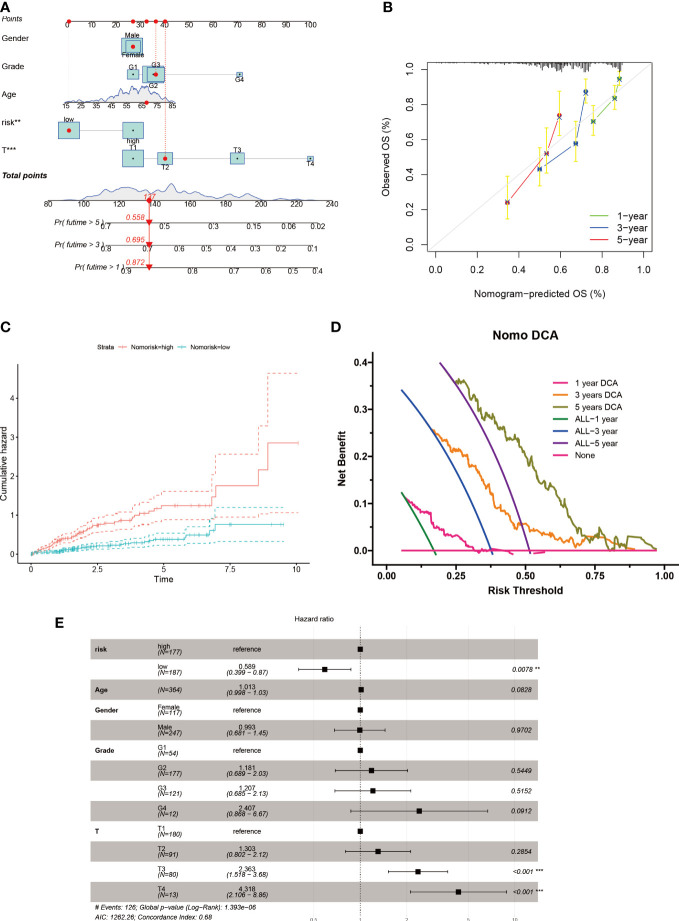
Nomogram construction for HCC patients. **(A)** Nomogram established based on risk scores and clinicopathological features. **(B)** Calibration plot validates the reliability of this nomogram. **(C)** Survival probability was represented by the cumulative hazard curve over time. **(D)** DCA curves of the nomogram for survival status of HCC patients. **(E)** Multivariable Cox regression analyses of the clinical features as well as risk score in HCC patients.

In order to account for the influence of clinical factors such as age, gender, and stage on tumor progression, we incorporated them into a nomogram (**Figure 6A**) along with the riskscore. The nomogram’s accuracy was verified using a calibration plot (**Figure 6B**) and cumulative risk curves that demonstrated an increasing survival risk for HCC patients with high scores (**Figure 6C**). A decision curve analysis revealed that this nomogram has long-term benefits for HCC patients and can serve as a reference tool for clinical decision-making (**Figure 6D**). A forest plot showed that T stage and riskscore were the primary factors affecting the nomogram (**Figure 6E**).

### 3.7 SPGs expression in HCC

**Figure 7:**
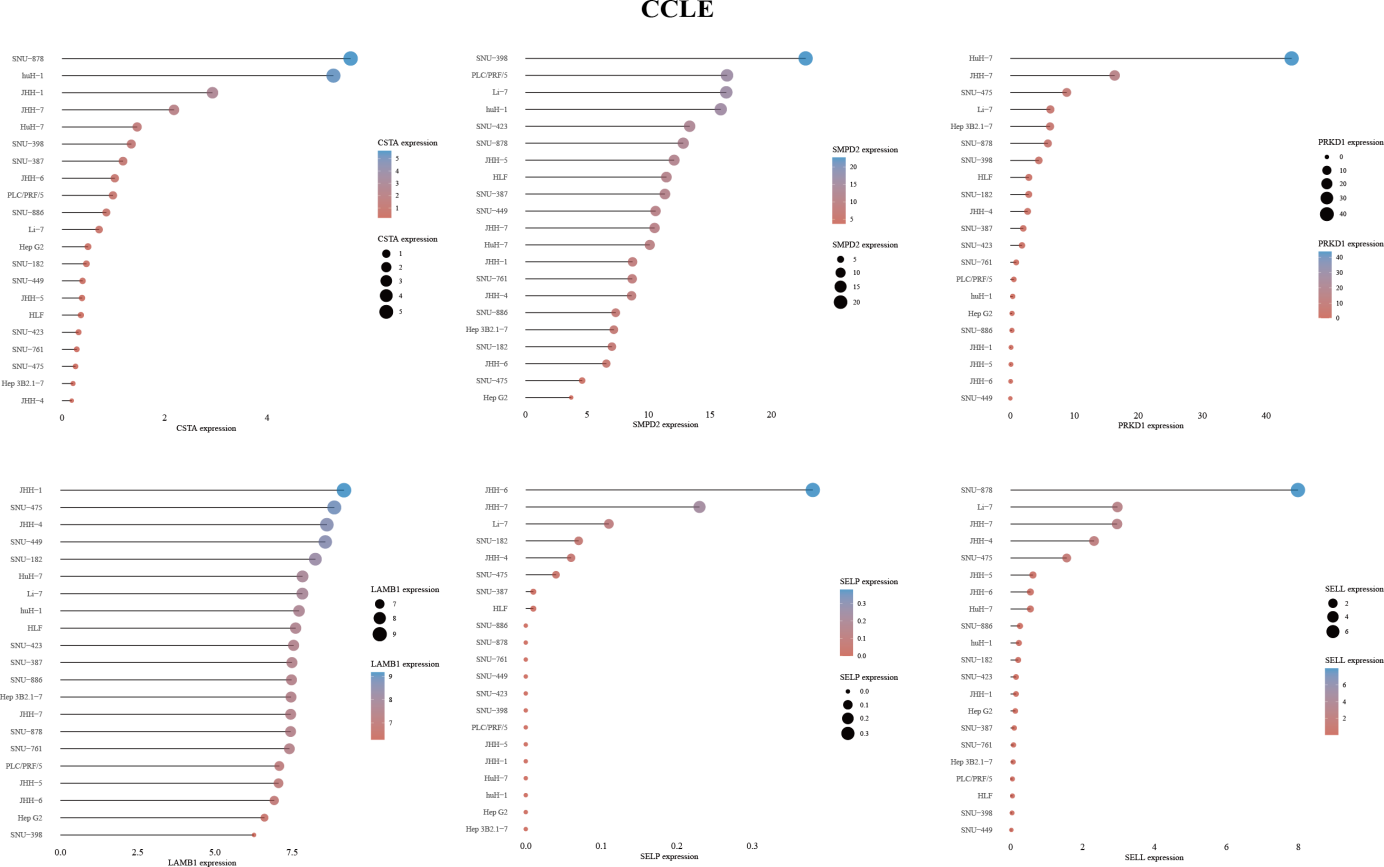
6-SPGs expression patterns in HCC cell lines based on CCLE database.

**Figure 8:**
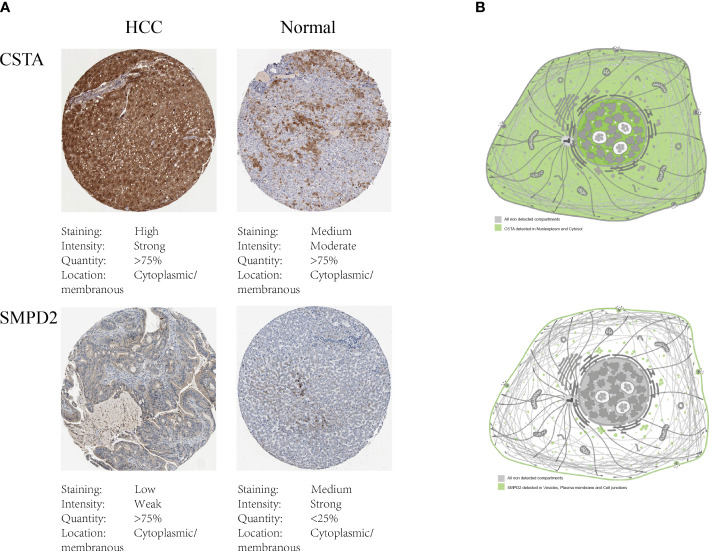
Protein expression of SPGs. **(A)** IHC results of CSTA and SMPD2. **(B)** The subcellular distribution of CSTA and SMPD2 proteins in human cell lines.

To investigate the impact of key metabolic genes of SPGs on the development of HCC, we first screened for the optimal HCC cell lines for our experiments by evaluating the expression levels of SPGs. The Huh7 cell line was chosen due to its high expression levels of SPGs that closely matched the identified risk factors (**Figure 7**). As CSTA and SMPD2 were identified as having the highest weight in the risk model, we hypothesized that they may play a major role in HCC development. The HPA portal was used to examine the expression levels of CSTA and SMPD2 proteins in HCC tissues. As shown in **Figure 8A**, the results of IHC revealed that CSTA protein levels were significantly higher in HCC tissues compared to normal tissues, with stronger staining intensity. Interestingly, despite the low staining of SMPD2 in tissues, we found that SMPD2 protein expression was detected in over 75% of HCC tissues, significantly higher than the 25% found in normal samples. In addition, **Figure 8B** highlights the localization of CSTA and SMPD2 proteins in the cell, with CSTA present in the nucleoplasm and cytosol and SMPD2 found in vesicles, the plasma membrane and cell junctions, suggesting different functions between CSTA and SMPD2.

### 3.8 Prevention of SPG damages HCC cell migration ability

**Figure 9:**
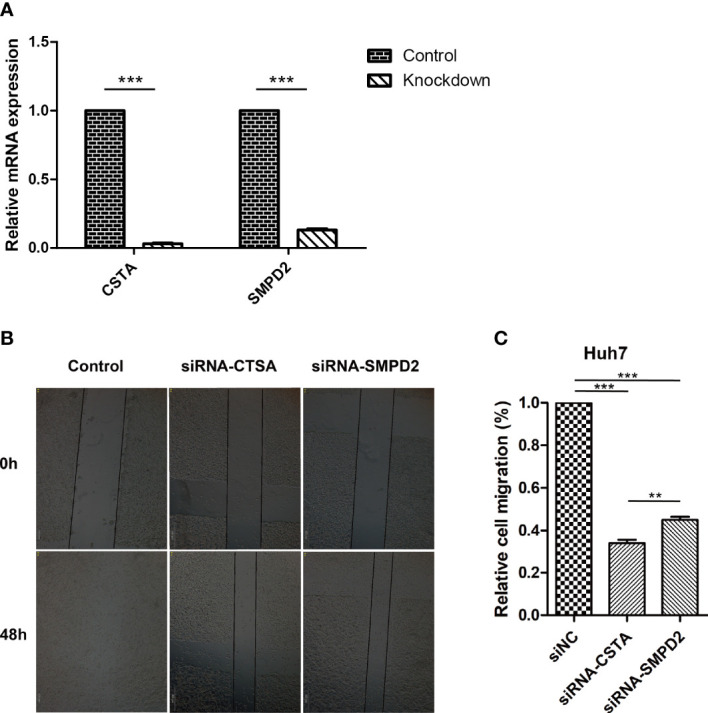
Inhibition of SPGs impairs HCC cells migration ability. **(A)** realtime-PCR. **(B)** Wound-healing assay. (**P < 0.01, ***P < 0.001).

The recurrence of cancer through metastasis is a significant contributor to poor outcomes in HCC (40). The spread of tumor cells to other parts of the body makes treatment more difficult (41). To understand the impact of silencing specific genes on the migration of HCC cells, we used siRNA to study this effect (**Figure 9A**). Our results, shown in **Figure 9B**, indicate that the silencing of CSTA and SMPD2 genes significantly decreased the migration of Huh7 cells. Furthermore, the impact of CSTA on the mobility of Huh7 cells was greater than that of SMPD2.

### 3.9 Inhibition of SPGs makes HCC cells sensitive to lapatinib drug

**Figure 10:**
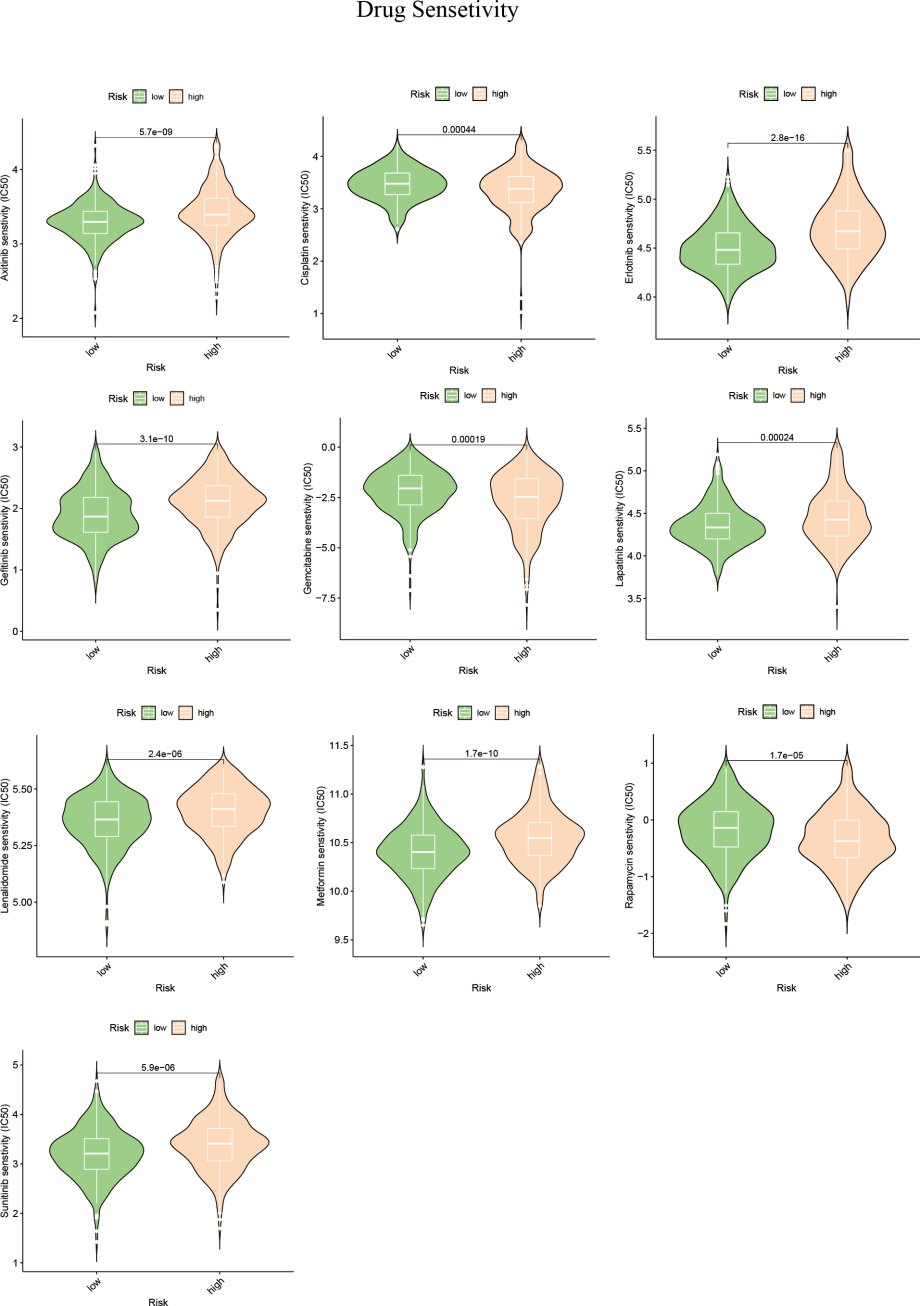
Drug predictions based on SPGs risks core. The “pRRophetic” R package was used to estimate the median inhibitory concentration (IC50) of small molecule drugs.

**Figure 11:**
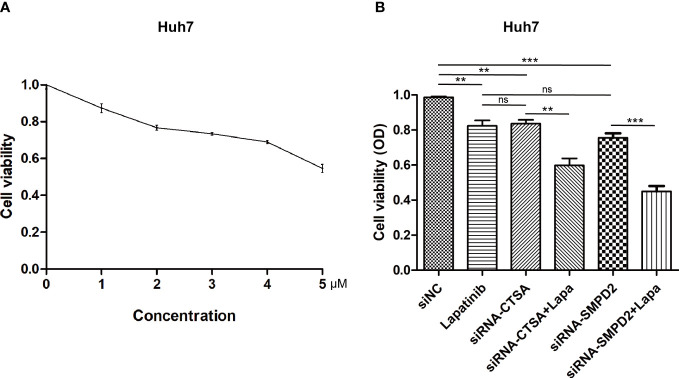
Genetic Inhibition of SPGs Sensitizes HCC cells to lapatinib drug. **(A)** Cytotoxicity at different doses of lapatinib. **(B)** Cell viability was measured using the CCK-8 assay at 48 hours after treatment with siRNA of CSTA/SMPD2, combined with or without 3μM lapatinib in Huh7 cells (ns, not significant; p > 0.05; **p < 0.01; ***p < 0.001).

Alterations in the levels of certain genes involved in sphingolipid metabolism may have a significant impact on the effectiveness of chemotherapy (42, 43). Using gene expression data from different risk subgroups, we utilized the pRRophetic package to predict variations in the sensitivity to clinical antitumor drugs across different risk groups (**Supplementary Table S2**; **Figure 10**). Additionally, we employed the BEST database to predict the effects of immunotherapy (**Supplementary Figure S3**). Our findings indicate that HCC patients with high expression levels of sphingolipid metabolism genes (SPGs) may be more responsive to CAR-T treatment. Furthermore, the level of SPGs exhibited good predictive power for response to immunotherapy (AUC = 0.712). Among the drugs tested, lapatinib was found to be more effective in low-risk groups (**Figure 10**). As CSTA and SMPD2 were identified as key SPGs, we investigated the effects of silencing these genes on the sensitivity of HCC cells to lapatinib (**Figure 11A**). Our results revealed that downregulation of CSTA or SMPD2 through siRNA transfection significantly increased the sensitivity of Huh7 cells to lapatinib (**Figure 11B**).

### 3.10 Investigating the distribution of SPGs using single-cell analysis

**Figure 12:**
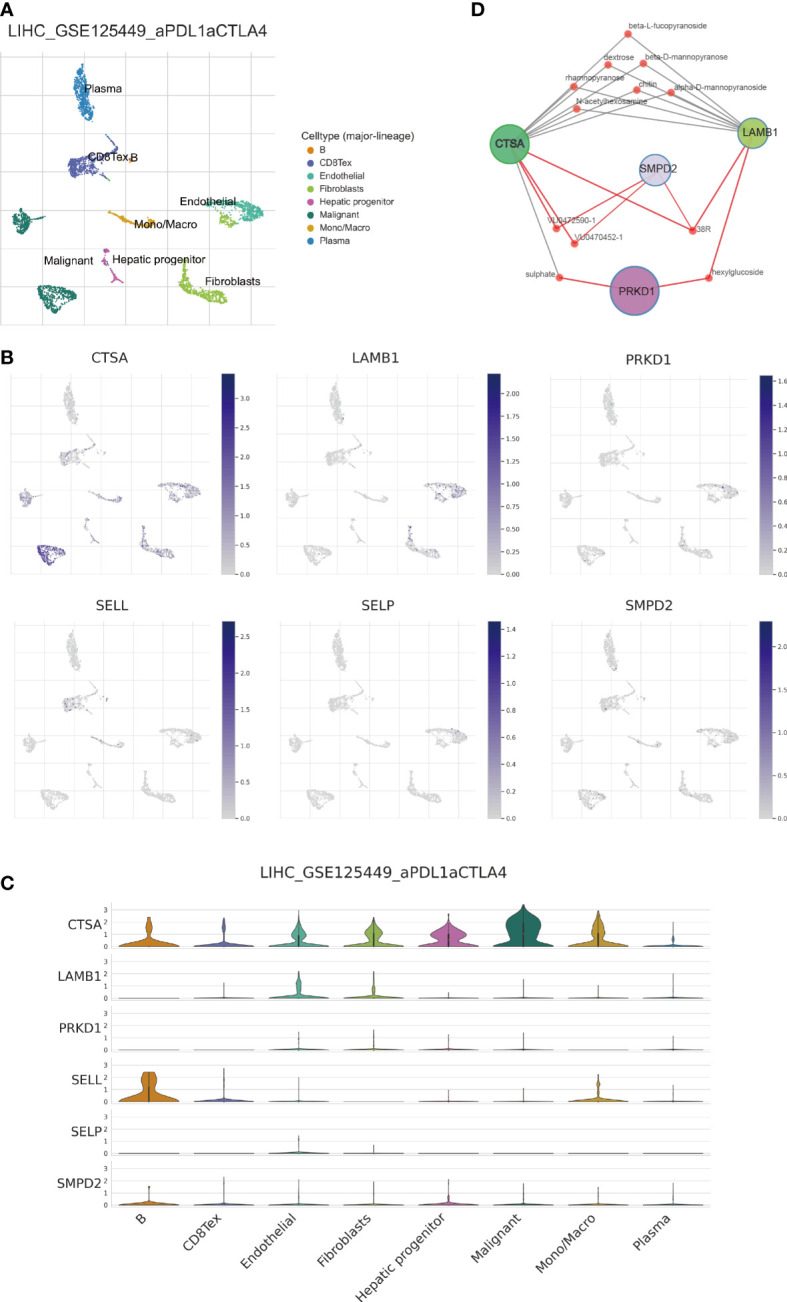
Single-cell analysis of 6-SPGs expression patterns. **(A)** GSE125449 annotation of all cell types and percentage of each type. **(B, C)** Percentages and expressions of 6-SPGs in HCC tissues. **(D)** Prediction of targeted drugs for four highly expressed SPG genes.

Next, we performed single-cell analysis to investigate the expression levels of SPGs in various immune cells from HCC patients. Utilizing the GSE125449 dataset, we identified 8 major cell types (**Figure 12A**) and visualized the expression levels of 6 SPGs using the single-cell dataset GSE125449 from the TISCH database (**Figures 12B, C**). As previously observed, CSTA was primarily expressed in malignant cells, while SELL was primarily expressed in CD8T and B cells. These findings have the potential to inform the development of targeted gene therapy strategies for specific types of cells. Additionally, we further explored potential gene-targeted drugs through Quartata Web. The red node links represent predicted protein-chemical interactions, and gray links represent known protein-chemical interactions. The color of the red nodes represents a chemical (**Figure 12D**).

## 4 Discussion

Hepatocellular carcinoma (HCC) is a challenging malignancy that poses a significant threat to human life (1). The complexity of the molecular mechanisms underlying HCC makes it difficult to improve the prognosis. Single-targeted pathway or drug therapy alone is not sufficient to effectively improve the prognosis of HCC (44–46). Therefore, using multiple genes in constructing predictive models may be a more effective approach. However, there is currently a lack of sufficient biomarkers for this purpose. It is important to identify more biomarkers to improve the accuracy of predictive models for early intervention in HCC.

Cell membranes are composed of various lipids, including sphingolipids, which play a critical role in maintaining the structural integrity of the barrier and regulating fluidity (12). Nutrient metabolism is critical to the survival of tumor cells (46). Sphingolipids are members of a class of lipids. Additionally, sphingolipids act as secondary messengers in cell signaling and are involved in regulating various biological processes (47). In recent years, scientists have made significant strides in identifying and cloning the metabolic enzymes that control sphingolipid content. The activity of these enzymes can have a significant impact on how cancer develops and how it responds to treatment (48). Research suggests that sphingolipids may contribute to the development of various types of cancer, including HCC. The tumor microenvironment (TME) is a complex and dynamic system composed of diverse cells and non-cellular components, such as TAM, T cells, and B cells (49–54). These elements interact with cancer cells to create the TME, which can have a significant impact on treatment outcomes. Increasing evidence suggests that the diversity of the TME is responsible for variations in treatment outcomes (55–59).

We used 28 SPGs to classify HCC patients into four subtypes, with the clusterD subtype having the worst prognosis. This subtype was characterized by higher levels of SPG expression and increased immune infiltration involving MDSC cells and Treg cells with immunosuppressive properties, as shown in **Figure 3**. The poor prognosis associated with HCC may be partly attributed to the immunosuppressive microenvironment promoted by SPGs. Additionally, macrophages play a crucial role in innate immunity, and their infiltration levels are often considered a marker of chronic inflammation. Macrophages in cancer have both pro- and anti-tumor activities, with macrophage M1 and M2 playing important roles. Our data in **Figures 5B, C** showed a significant and positive association between macrophage M2 infiltration and the risk score. Further analysis revealed that CSTA, LAMB1, and PRKD1 may be critical factors that induce macrophage M2 infiltration, as shown in **Figure 5F**. Therefore, we hypothesized that HCC cells may promote macrophage M2 infiltration, but not M1 infiltration, by upregulating these sphingolipid-related genes. Blocking the aggregation of macrophage M2 or targeting these SPGs may be potential interventions to inhibit HCC progression.

Sphingolipids are known to play a crucial role in tumor growth and its interactions with various pathways related to cancer (60–65). However, many genes associated with sphingolipids remain poorly understood and have not been extensively researched as potential therapeutic targets in clinical settings (66, 67). We identified six genes that form a strong risk score signature. To further validate our findings, we suppressed the expression of SMPD2 in Huh7 cells, as shown in **Figure 9A**, and conducted a wound healing assay, as shown in **Figure 9B**. These results support the idea that SMPD2 may be a potential therapeutic target for HCC, as inhibiting its expression may impede the migration and survival of HCC cells.

In subsequent experiments, SMPD2 and CSTA were chosen as key factors due to their high importance in the risk model score. It was found that silencing SMPD2 and CSTA using siRNA significantly decreased the migration ability and increased apoptosis in Huh7 cells (**Figure 9**). Previous research has shown that changes in sphingolipid metabolism can greatly affect the tumor cells’ sensitivity to chemotherapy. By analyzing drug sensitivity, we discovered that low-risk HCC patients were more responsive to lapatinib (**Figure 10**). As a result, we aimed to confirm whether inhibiting SMPD2 or CSTA enhances the sensitivity of liver cancer cells to lapatinib (**Figure 11B**). Additionally, it is worth noting that SMPD2 is mainly located on lipid droplets and plasma membranes (**Figure 8B**), and lipid droplet formation is known to confer resistance of HCC cells to chemotherapy (43). This may partly explain why lapatinib increases the sensitivity of HCC cells to SMPD2 inhibition. Overall, it appears that cell survival is more affected by SMPD2, while migration is more affected by CSTA.

The use of gene expression profiling to classify tumor samples has been well-established in previous research (68–74). Building on this approach, we classified clinical cohorts of HCC patients based on the expression levels of six specific genes associated with sphingolipids. This classification revealed significant differences in prognostic outcomes, indicating that our genetic model can effectively predict patient prognosis and response to treatment options such as immunotherapy and chemotherapy. This information can aid clinicians in making treatment decisions for HCC patients. Furthermore, our analysis using DCA curves showed that patients at 1, 3, and 5 years could benefit from nomograms constructed using these six genetic features (**Figure 6D**).

## 5 Conclusion

Our study created a six-gene signature and prediction models that could aid healthcare providers in selecting individualized treatment options for HCC patients. Additionally, it uncovered the connection between sphingolipid-related genes and the immune microenvironment, offering a new approach for immunotherapy. By focusing on crucial sphingolipid genes, such as SMPD2 and CSTA, the sensitivity of HCC to anti-tumor therapy may be enhanced.

## Data availability statement

The datasets presented in this study can be found in online repositories. The names of the repository/repositories and accession number(s) can be found in the article/**Supplementary Material**.

## Author contributions

XZ and YL conceived the study. XZ, JZ, JL, ZX, HW, QG, HJ, YQ, LF, and YL drafted the manuscript. JZ, XZ, CT, and WL performed the literature search and collected the data. XZ, ZX, JL, and HW analyzed and visualized the data. ZX and HW completed all experiments. JZ, WL and YL helped with the final revision of this manuscript. All authors reviewed and approved the final manuscript.

## Supplementary material

Supplementary Figure 1 Genes’ Sequences of primers.

Supplementary Figure 2 Immune infiltration in SPGcluster.

Supplementary Figure 3 Immunotherapy prediction *via* 6-SPGs. **(A)** PRF survival between high-and low- SPGs subgroups. **(B)** Prediction sensitivity to immunotherapy response *via* 6-SPGs.
