# Supplementary figures and images for: Prognostic signatures of sphingolipids: Understanding the immune landscape and predictive role in immunotherapy response and outcomes of hepatocellular carcinoma
Source: Front Immunol. 2023 Mar 17;14:1153423. doi: 10.3389/fimmu.2023.1153423 (PMC10063861; doi:10.3389/fimmu.2023.1153423)

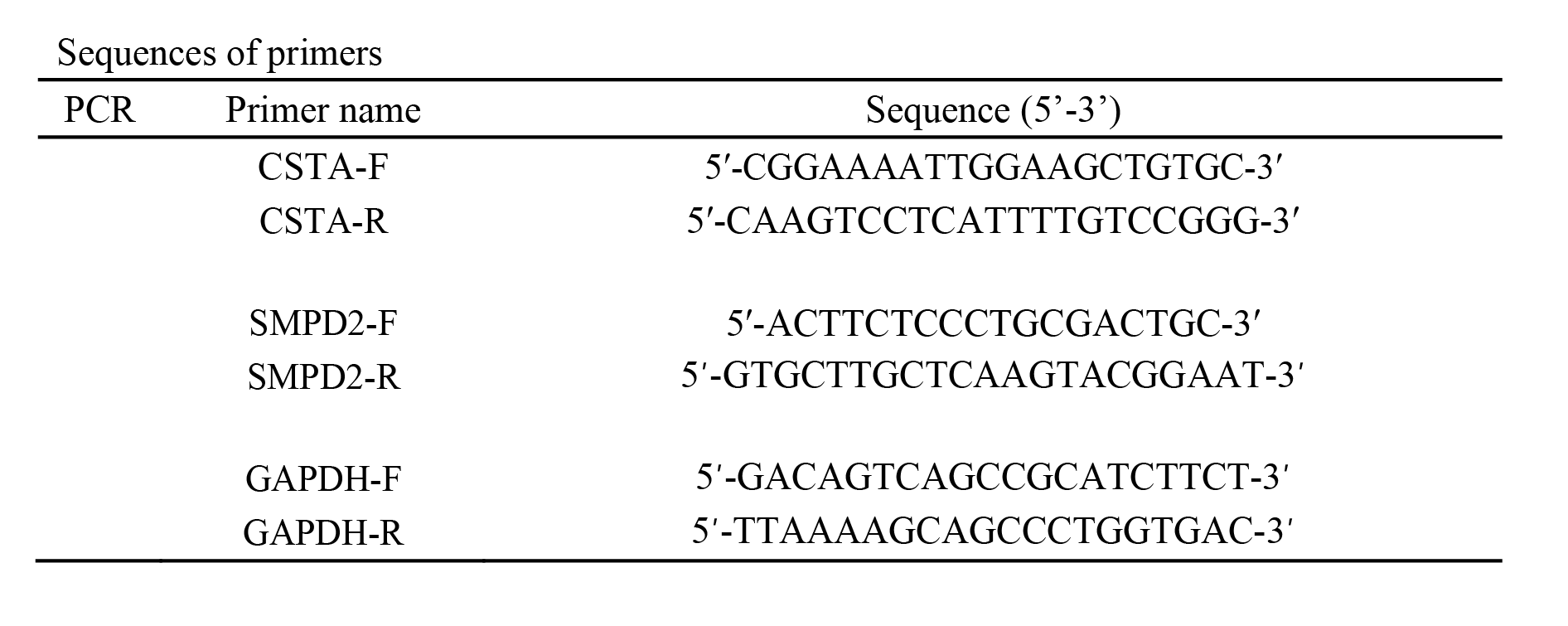

Supplement: Supplementary Figure 1 — Genes’ Sequences of primers. [file Image_1.tif]

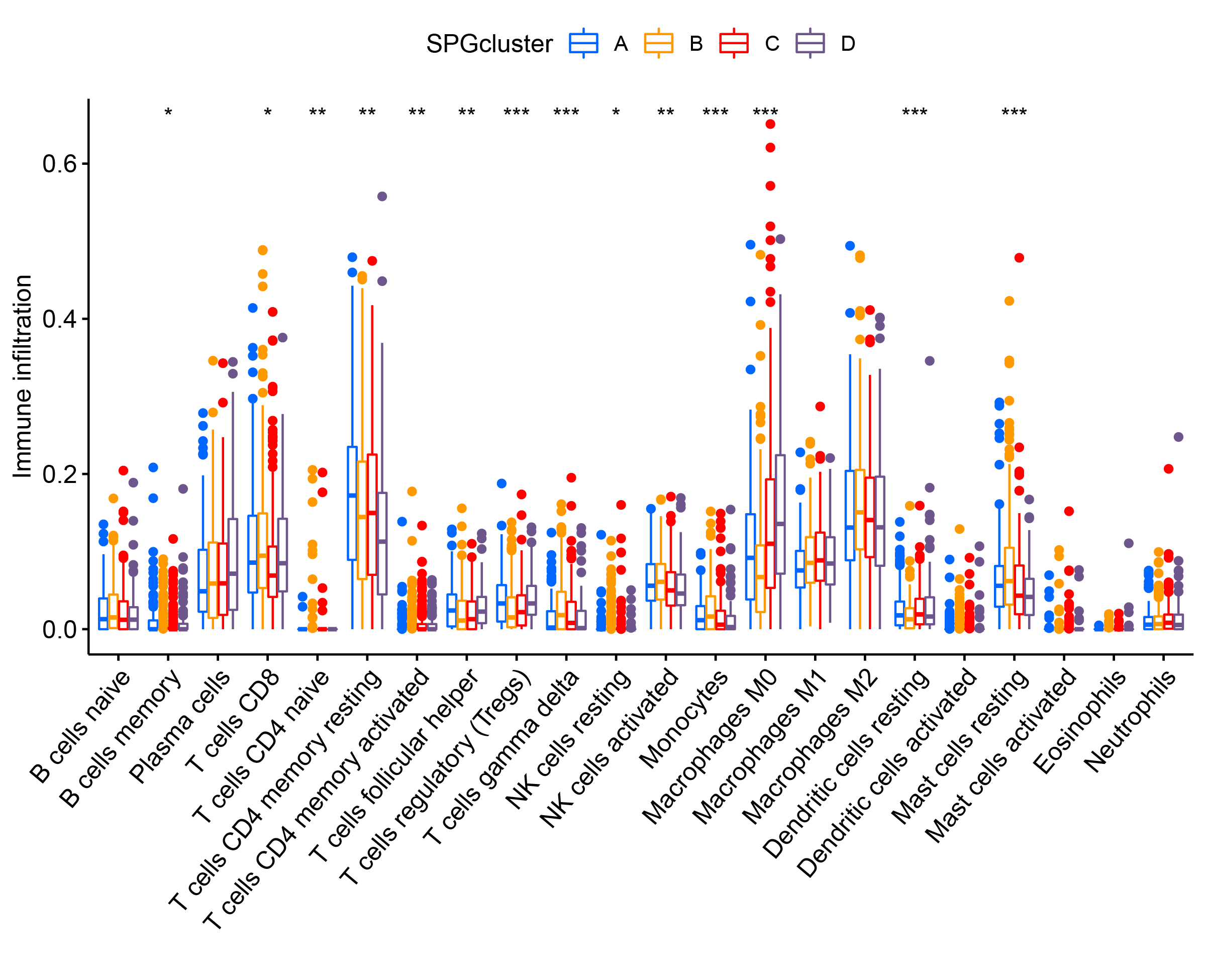

Supplement: Supplementary Figure 2 — Immune infiltration in SPGcluster. [file Image_2.tif]

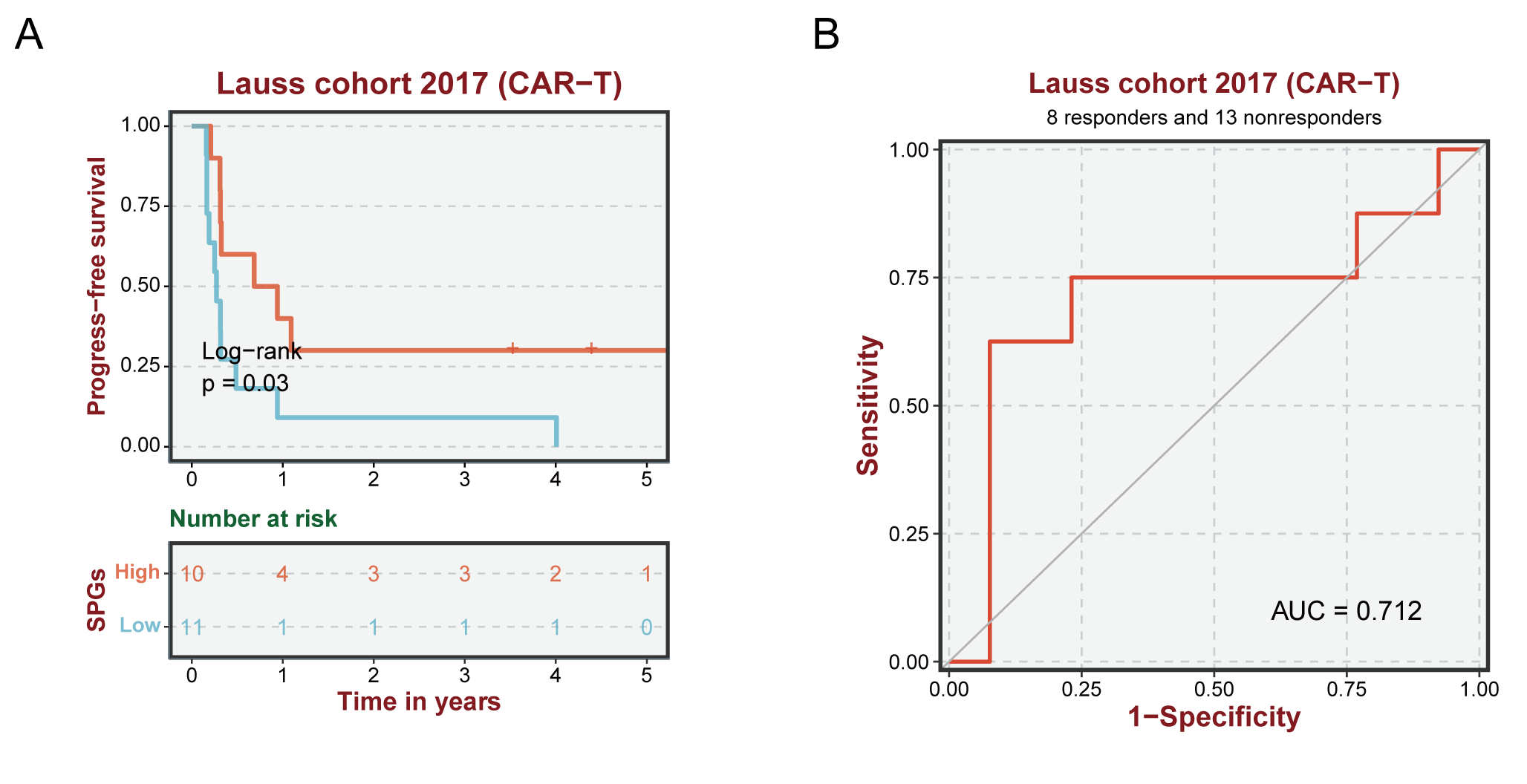

Supplement: Supplementary Figure 3 — Immunotherapy prediction via 6-SPGs. (A) PRF survival between high-and low- SPGs subgroups. (B) Prediction sensitivity to immunotherapy response via 6-SPGs. [file Image_3.tif]
